# Prevalence of human papillomavirus in young Italian women with normal cytology: how should we adapt the national vaccination policy?

**DOI:** 10.1186/1471-2334-13-575

**Published:** 2013-12-06

**Authors:** Donatella Panatto, Daniela Amicizia, Elisabetta Tanzi, Silvia Bianchi, Elena Rosanna Frati, Carla Maria Zotti, Piero Luigi Lai, Angela Bechini, Stefania Rossi, Roberto Gasparini

**Affiliations:** 1 Department of Health Sciences, Genoa University, Via Pastore, 1, Genoa 16132, Italy; 2 Department of Biomedical Sciences for Health, Milan University, Milan, Italy; 3 Department of Public Health and Paediatrics Sciences, Turin University, Turin, Italy; 4 Department of Health Sciences, Florence University, Florence, Italy; 5 Department of Molecular and Developmental Medicine, Siena University, Siena, Italy

**Keywords:** Papillomavirus, HPV, Cervical cancer prevention, Vaccination, HPV epidemiology, Young women

## Abstract

**Background:**

Human Papillomavirus (HPV) is the most common sexually transmitted infection. In Italy, HPV vaccination is now offered free of charge to 12-year-old females. However, some regional health authorities have extended free vaccination to other age-groups, especially to girls under 18 years of age. We conducted a multicentre epidemiological study to ascertain the prevalence of different genotypes of HPV in young Italian women with normal cytology, with the aim of evaluating the possibility of extending vaccination to older females.

**Methods:**

The study was performed in 2010. Women aged 16–26 years with normal cytology were studied. Cervical samples were analyzed to identify the presence of HPV by PCR amplification of a segment of ORF L1 (450 bp). All positive HPV-DNA samples underwent viral genotype analysis by means of a restriction fragment length polymorphism assay.

**Results:**

Positivity for at least one HPV genotype was found in 18.2% of the 566 women recruited: 48.1% in the 16–17 age-class, 15.4 in the 18–20 age-class, 21.9% in the 21–23 age-class, and 15.5% in the 24–26 age-class; 10.1% of women were infected by at least one high-risk HPV genotype. HPV-16 was the most prevalent genotype. Only 4 (0.7%), 4 (0.7%) and 3 (0.5%) women were infected by HPV-18, HPV-6 and HPV-11, respectively. Of the HPV-DNA-positive women, 64.1% presented only one viral genotype, while 24.3% had multiple infections. The HPV genotypes most often involved in multiple infections were high-risk. A high prevalence was noted in the first years of sexual activity (48.1% of HPV-DNA-positive women aged 16–17 years); HPV prevalence subsequently declined and stabilized.

The estimate of cumulative proportions of young women free from any HPV infection at each age was evaluated; 93.3% and 97.1% of 26 year-old women proved free from HPV-16 and/or HPV-18 and from HPV-6 and/or HPV-11, respectively.

**Conclusions:**

Our findings confirm the crucial importance of conducting studies on women without cytological damage, in order to optimise and up-date preventive interventions against HPV infection, and suggest that vaccinating 26-year-old females at the time of their first pap-test is to be recommend, though this issue should be further explored.

## Background

Many types of human papillomavirus (HPV) are known. Of these, about 50 have a high tropism for the ano-genital mucosa and are sexually transmitted. According to the most recent classification of the International Agency for Research on Cancer (IARC) 12 genotypes are defined as high-risk (HR) oncogenic (HPV-16, 18, 31, 33, 35, 39, 45, 51, 52, 56, 58, and 59). Moreover, a probable oncogenic risk has been assigned to genotype 68, and a possible oncogenic risk to the types: HPV-26, 53, 66, 67, 70, 73, 82, 30, 34, 69, 85, and 97. Many low-risk (LR) genotypes that cause benign lesions and warts are also known (HPV 6, 11, 28, 32, 40, 42, 43, 44, 54, 55, 57, 61, 62, 71, 72, 74, 81, 83, 84, 86, 87, and 89) [1].

After zur Hausen’s discovery of the association between persistent infection by oncogenic types of HPV and cervical cancer [2], epidemiological studies and experimental research into the development of preventive vaccines began. Since that time, our knowledge of the virus in subjects with HPV-associated lesions has increased, revealing the great complexity of the ecosystem of this pathogen [3-5].

The prevalence of HPV in subjects with lesions displays a different distribution by region and age [6-9]. The first epidemiological data were obtained in the United States [10-14]. Subsequently, many observations were collected in the rest of the world [15,16]. Several studies have also been conducted in Europe and in Italy [17-24].

So far, two HPV vaccines have been licensed: a bivalent vaccine (Cervarix® GlaxoSmithKline Biologicals S.A.) and a quadrivalent vaccine (Gardasil® Merck and Co). Both have a prophylactic indication and are able to prevent pre-cancerous lesions and cancers due to persistent infection by HPV-16 and HPV-18. Furthermore, both vaccines have been shown to elicit cross-protection against other high-risk HPV types [25,26]. Gardasil® also offers protection against genital warts and low-risk lesions caused by HPV-6 and HPV-11.

The epidemiological data collected worldwide constitute the starting point for vaccination strategies. However, in order to draw up optimal vaccination policies, it is crucial to know the prevalence of the different HPV types in the overall population, and not only in subjects with lesions or cancer. Indeed, such knowledge is essential to predicting the expected impact and monitoring the actual impact of HPV immunisation. For this reason, several extensive studies on women with normal cytology have been conducted [7,27-29].

The World Health Organization and the European Centre for Disease Prevention and Control recommend HPV vaccination and indicate young females (aged 10–14 years) as the target population [30,31]. In Europe, almost every country offers vaccination free of charge to females aged 11–12 years [32]. Since 2008, HPV vaccination has been offered free of charge to 12-year-old females in Italy, and 70.6% of eligible subjects belonging to the first vaccinated cohort (females born in 1997) have been immunized with at least one dose [33].

Two years after the start of HPV vaccination, we conducted a multicentre epidemiological study to ascertain the prevalence of different genotypes of HPV in young Italian women with normal cytology, with a view to evaluating the possibility of extending vaccination – for instance by offering free vaccination to 26-year-old females.

## Methods

The study protocol, which was used by all research units, was approved by the Ethics Committee of the Local Healthcare Unit (LHU) in Genoa, Italy.

The study was performed in 2010 and enrolment was carried out during the first six months of the year.

### Study population

The study population was recruited in three cities in northern Italy (Turin, Milan and Genoa).

The study protocol involved enrolling all consecutive eligible young women aged 16–26 years who spontaneously accessed gynaecology centres of the LHUs for medical consultations from 1^st^ January to 30^th^ June 2010. We chose this age-group because it is potentially the best target for vaccination. Written informed consent was obtained from every participant. Women were considered eligible for enrolment if they were sexually active, were not pregnant, had not been vaccinated against HPV and had no previous history of cervical abnormalities.

All participants underwent a Pap smear and HPV-DNA test. Subsequently, only women with normal cytologically negative Pap smears were studied, as the aim of the study was to evaluate the prevalence of HPV genotypes in healthy young women without any HPV lesions.

Women with a positive pap smear were excluded from the study. They were followed up by the gynaecology centres of the LHUs or by their own gynaecologists and were treated in accordance with the Italian guidelines [34]. No data on these women were supplied to the researchers involved in this study.

### Sample collection

Cervical samples were collected by means of a spatula, immersed and rinsed in a vial containing 20 ml of PreservCyt® solution (ThinPrep Pap Test, Hologic, Italy) and stored at room temperature (RT) until processing. All samples were analysed in the molecular laboratory of the Department of Biomedical Sciences for Health – University of Milan.

Ten millilitres of each PreservCyt® solution containing cervical cells was centrifuged at 3800 g for 10 min at RT. Cellular pellets were resuspended in 1 ml Phosphate Buffered Saline (PBS) and stored at −20°C until molecular analyses were carried out.

### DNA extraction and HPV detection

DNA was extracted from cervical samples by means of a commercial method (NucliSENS*®* EasyMAG*®*, bioMérieux, Lyon, France) according to the manufacturer’s instructions. The concentration and purity of the DNA extracted were evaluated by means of a spectrophotometer (Thermo Scientific NanoDrop 2000; Thermo Fisher Scientific Inc., Wilmington, DE). DNA integrity was assessed by amplification of a 268 base pair (bp) fragment in the ubiquitous β-globin gene by using the primer pair PCO_4_ and GH_20_[35].

HPV DNA was detected by PCR amplification of a 450 bp segment of ORF L1 by using the degenerate primer pair ELSI-f and ELSI-r, as previously described [36]. Each PCR run included positive controls (DNA extracted from HPV 16-positive cells, CaSki) and negative (water) controls. The amplification products were visualized by means of electrophoresis analysis on 2% agarose gels containing ethidium bromide (0.5 mg/ml). Amplified product bands were compared with molecular weight standards (DNA Molecular Weight, Marker 100, Sigma-Aldrich, St. Louis, MO).

### Restriction fragment length polymorphism (RFLP) genotype analysis

All amplified fragments were subjected to viral genotype analysis by RFLP capable of identifying all HR, probable/possible HR, and LR genotypes of the alpha genus according to the new IARC classification system [1,37].

Amplified products (1 g each) were added to three different digestion solutions, each containing 1U of either *Rsa*I, *Dde*I or *Hae*III (New England BioLabs, Ipswich, MA) restriction enzymes diluted in their respective buffers for 1 h at 37°C. The digestion products were identified following separation in 3% agarose gels, and restriction patterns were compared with appropriate standards (DNA Molecular Weight, Marker 100 + 20, Sigma-Aldrich, St. Louis, MO). The pattern of fragments generated by the three restriction enzymes enabled the genotype to be identified [38,39].

Samples displaying complex or undetermined RFLP patterns were retested. Samples that could not be assigned with confidence to a certain type after two consecutive analyses were classified as “not typing” (NT).

### Statistical analysis

The prevalence of HPV-infected cases, together with their 95% Confidence Intervals (CI), was described by age-class and city of residence, and the Chi-square test was used to analyze the differences (a two-sided p-value < 0.05 was considered statistically significant). The subjects enrolled were broken down into 4 age-classes: 16–17, 18–20, 21–23 and 24–26 years old. In addition, the percentage of infections, broken down by risk-group in the different age-classes, was calculated on considering the total number of infections detected in each age-class.

The Kaplan-Meier method was used to estimate the cumulative proportions of young females who were free from HPV infection at each age, and graphic descriptions of all HPV genotypes and different HPV genotypes were drawn up. All analyses were performed with the Statistical Package for Social Sciences, for Windows, version 16.0 (SPSS, Inc., Chicago, Illinois).

## Results

A total of 650 women who met the inclusion criteria were invited to participate in the study; 16 declined. As few women refused to participate, the reasons for refusal were not investigated.

Of the 634 young women who agreed to take part in the study, 566 with normal cervical cytology were recruited. The 68 who were excluded had a positive Pap smear. In Milan, Genoa and Turin 281, 156 and 129 subjects were enrolled, respectively. Table 1 shows the demographic characteristics of the study population.

**Table 1:** Demographic characteristics of the study population

**Age-class (years)**	**Milan**	**Genoa**	**Turin**	**Total**
**16-17**	2	25	0	27
**18-20**	247	70	59	376
**21-23**	17	41	47	105
**24-26**	15	20	23	58
**Total**	281	156	129	566

The mean age of the participants on enrolment was 19.7 years (SD = 2.4). Positivity for at least one HPV genotype was found in 103 women (18.2%; 95% CI 10.7-25.6). In Milan, Genoa and Turin, 16.0% (95% CI 11.3-20.8), 24.4% (95% CI 17.8-31.9) and 15.5% (95% CI 9.3-22.9) of women, respectively, were HPV-positive. No statistically significant differences emerged among the cities of residence (p = 0.064). Among the 103 HPV-DNA-positive women, the samples from 12 subjects (11.6%) were NT.

**Figure 1:**
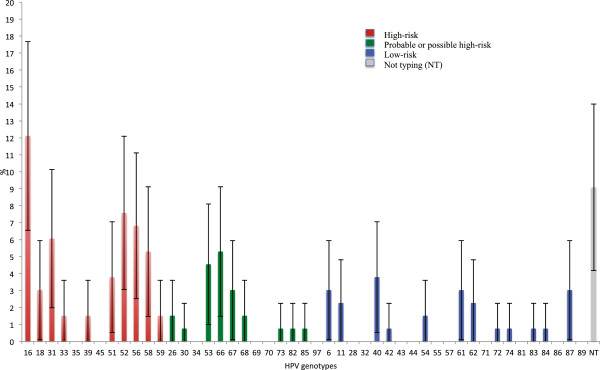
Percentages of all genotypes detected on considering the total number of infections.

Regarding high-risk HPV types, fifty-seven women (55.3%) were infected by at least one high-risk HPV genotype. HPV-16 was the most prevalent genotype, being detected in 16 women (15.5%). Only 4 subjects (3.9%) were infected by HPV-18. Sixty-six subjects (64.1%) presented only one viral genotype, while 25 (24.3%) had multiple infections. Globally, 132 HPV infections were detected in the 103 HPV DNA-positive women. Figure 1 shows all the HPV genotypes detected and highlights the relevance of the various genotypes in relation to the total number of infections. With regard to high-risk genotypes HPV-16 was most frequently detected, followed by HPV-52 and HPV-56.

Regarding the importance of the different genotypes on considering the infection type (single or multiple) we found that most of the single infections were caused by high-risk or probable/possible high-risk HPV genotypes (74.2%). Concomitant HPV-16 and HPV-18 infections were not found.

**Figure 2:**
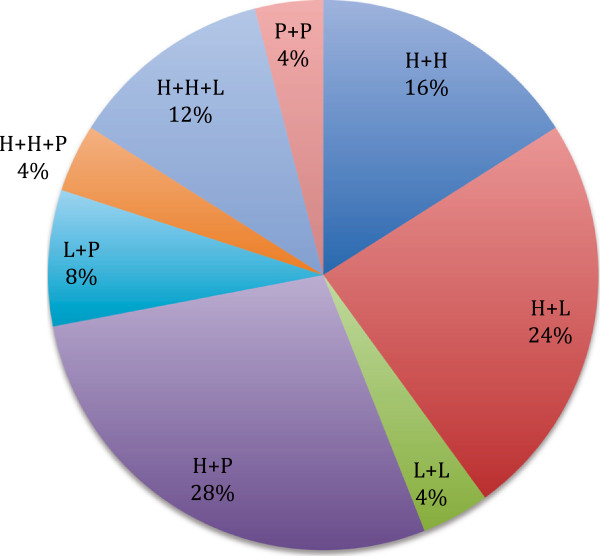
**Distribution of multiple infections on the basis of HPV combinations.** H+H: double infection by two high-risk HPVs; H+L: double infection by one high- and one low-risk HPVs; L+L: double infection by two low-risk HPVs; H+P: double infection by one high- and one probable/possible-risk HPVs; L+P: double infection by one low- and one probable/possible-risk HPVs; H+H+P: triple infection by two high-risk and one probable/possible-risk HPVs; H+H+L: triple infection by two high-risk and one low-risk HPVs; P+P: double infection by two probable/possible-risk HPVs.

Figure 2 shows the distribution of multiple infections on the basis of HPV combinations. It can be seen that the most frequent multiple infections were sustained by high-risk genotypes combined with probable/possible high-risk genotypes (28%). The most frequent HPV involved in multiple infections was HPV-16 (16%), followed by: HPV-52 (12%), HPV-53 (8%), HPV-58 (8%) and HPV-56 (6%).

Regarding low-risk HPV types, HPV-40 was the genotype most often detected. Only 4 (3.9%) and 3 (2.9%) women were infected by HPV-6 and HPV-11, respectively. Of 25 multiple infections, only one was caused exclusively by low-risk HPV genotypes (HPV-6 and HPV-11 together).

Table 2 shows the prevalence of subjects positive for at least one HPV type, broken down by age-class. The difference in prevalence among the different age-classes was highly significant (p < 0.0001). A particularly high prevalence was noted in the first years of sexual activity (48.1% of HPV-DNA-positive women in the 16–17 age-class); this was seen to decline and stabilize in the subsequent age-classes. Furthermore, a high prevalence of multiple infections was also observed in the 16–17 age-class; specifically, the distribution of the percentages of multiple infections on the basis of age was: 11.1% (95% CI 0–22.9) (16–17 age-class), 4.5% (95% CI 2.4-6.6) (18–20 age-class), 2.8% (95% CI 0–6.0) (21–23 age-class), and 3.4% (95% CI 0–8.1) (24–26 age-class).

**Table 2:** Prevalence of negative and positive subjects for at least one HPV type, broken down by age-class

	**HPV-DNA**		
**Age-class (years)**	**Negative**	**Positive**	**Total**
	**N° (%)**	**N° (%)**	**N° (%)**
	**95% CI**	**95% CI**	
**16-17**	14 (51.9)	13 (48.1)	27 (100)
	33.0-70.7	29.2-66.9	
**18-20**	318 (84.6)	58 (15.4)	376 (100)
	80.9-88.2	11.7-19.0	
**21-23**	82 (78.1)	23 (21.9)	105 (100)
	70.2-86.0	14.0-29.8	
**24-26**	49 (84.5)	9 (15.5)	58 (100)
	75.2-93.8	6.2-24.8	
**16-26**	463 (81.8)	103 (18.2)	566
	78.4-84.9	15.1-21.6	

**Figure 3:**
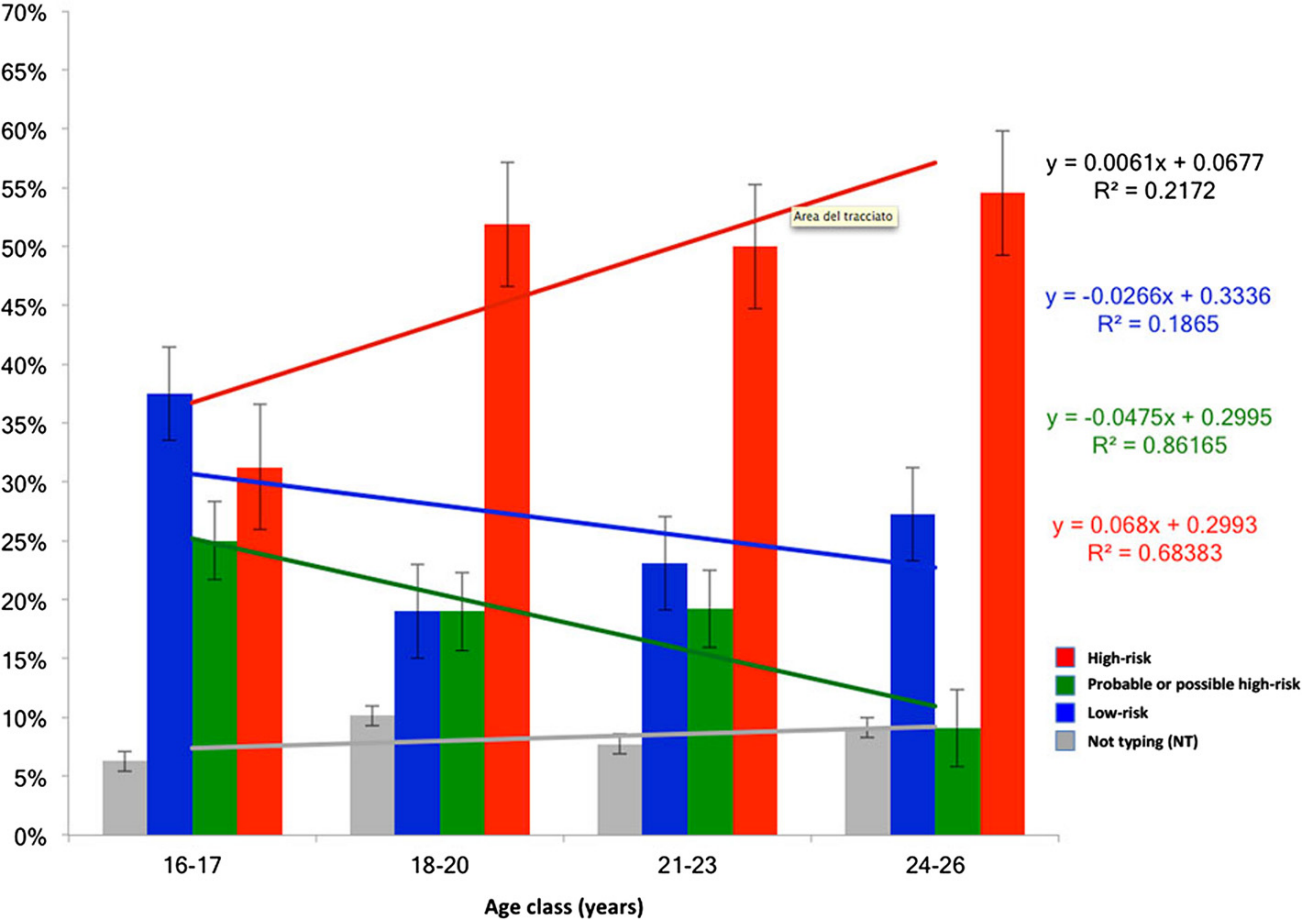
Percentages of infections broken down by risk-group in the different age-classes on considering the total number of infections detected in each age-class.

Figure 3 shows the percentage of infections broken down by risk-group in the different age-classes on considering the total number of infections detected in each age-class. The percentage of high-risk HPV infections was seen to increase with increasing age, while the percentages of the probable or possible high-risk and low-risk HPV infections decreased with increasing age.

**Figure 4:**
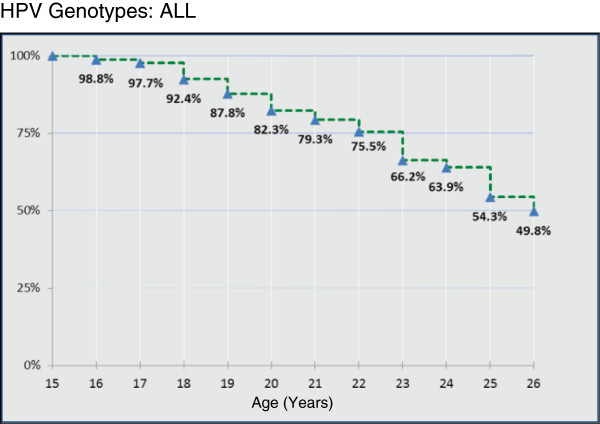
Estimated cumulative proportions of young females free from any HPV infection at each age.

Figure 4 shows the estimate of cumulative proportions of young women free from any HPV infection at each age.

**Figure 5:**
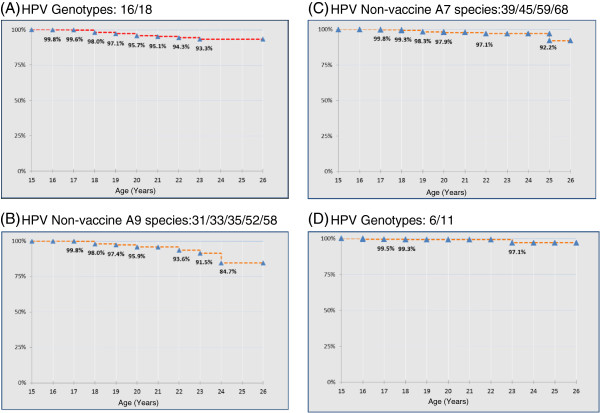
Estimated cumulative proportions of young females free from high-risk vaccine genotypes (HPV-16 and HPV-18) (A), free from high-risk non-vaccine genotypes (B and C) and free from low-risk vaccine genotypes (HPV-6 and/or HPV-11) (D) at each age.

Figure 5 shows the estimate of cumulative proportions of women free from high-risk vaccine genotypes (HPV-16 and HPV-18), free from high-risk non-vaccine genotypes and free from low-risk vaccine genotypes (HPV-6 and HPV-11) by age; 93.3% and 97.1% of 26 year-old women proved free from HPV-16 and/or HPV-18 and from HPV-6 and/or HPV-11, respectively.

## Discussion

Our study revealed that 18.2% of the sexually active females with normal cervical cytology were infected by at least one HPV genotype; this result is consistent with data obtained by other authors in women without lesions [26,28]. In 2007, de Sanjosé et al. performed a meta-analysis on worldwide HPV prevalence and genotype distribution in women with normal cytology. On examining the data on young (< 25 years old) European women, they found a prevalence of about 22%, which was similar to that reported by Bruni et al. in the same age-group of young European women [26,28].

A prevalence study on HPV infection in women aged 18–24 years was carried out in 2006–2007 in Italy. The authors found a prevalence of HPV of about 30% and, on considering only the high-risk genotypes, a prevalence of approximately 20%. These values are higher than ours. However, their study population included women with normal cytology and women with cytological lesions. On considering only the data from the women with negative cytology, the authors found a prevalence of about 20%, which is comparable to our findings [40].

We recorded a prevalence of high-risk HPV genotypes of 10.1%, while Clifford et al. found a prevalence of high-risk HPV genotypes of 5.2% in Europe. However, these authors considered a very large age-range, namely 15–74 years [6].

Regarding the relative importance of different high-risk HPV types, the 6 most important in our study were: HPV-16, -52, -56, -31, -51, -18. Our results are similar, but not identical, to those reported by other authors. In Europe in 2005, Clifford et al. reported the following high-risk HPV types: HPV-16, -31, -18, -56, -45 and −35 [6]; Bruni et al. reported the types HPV-16, -31, -18, -33, -52 and -51 [28], and de Sanjosé, who considered the 5 most important types, reported HPV-16, -31, -18, -33 and -35 [26]. Our results seem to confirm the growing importance of type 52 in Italy and Europe, as in other parts of the world (North America, Africa and Asia) [6,26,28]. Mollers et al., who assessed the prevalence of genital HPV infections in a large cohort of sexually active young women (16–29 years old) in the Netherlands, found that the most prevalent high-risk HPV types were HPV-16, -51 and -52. However, as these authors did not perform cytological analyses, their study population included healthy women and women with lesions [41].

Another study that provided robust baseline estimates of the prevalence and distribution of HPV types among a nationally representative sample of women (25–64 years old) in England, prior to any impact of the National HPV Immunisation Programme, indicated that the most frequent HPV genotypes among subjects with normal cytology were: HPV-16, -52, -31 and -45 [42]. These data were confirmed by subsequent findings obtained by the same authors in young females up to 24 years old [18]. Furthermore, in a recent Dutch study, HPV types 16, 52, 51 and 31 had the highest type-specific incidence rates among young females (18–29 years old) in the pre-vaccine era; the researchers did not consider the cytological status of the study sample [43].

Among the HPV genotypes recently defined as probable/possible high-risk, we most frequently identified type 66, while among low-risk viruses type 40 was most frequently identified.

Regarding multiple infections, which are associated with a higher risk of developing high-degree precancerous lesions, we found a higher prevalence (24.3%) than other authors [44,45]. For instance, in French women under 25 years of age undergoing routine gynaecological examinations, Monsonego et al. found a prevalence of 9.3%, although these women were not selected through the filter of normal cytological findings [46]. The lower prevalence observed by Monsonego et al. could be explained by the fact that the mean age of the French women was probably higher than that of the women in our sample [46].

In young women (20–29 years old) randomly selected from a population of Danish women, Nielsen et al. found a prevalence of multiple infections of 10.5% [47]. The difference between our data and those of Nielsen et al. could be explained by the fact that our subjects were younger than those examined by the Danish authors.

Furthermore, in our study, although the subjects recruited were not distributed homogeneously by age, the highest percentage of multiple infections (11.1%, 95% CI 0–23.0) was seen among 16-17-year-old subjects. This result is consistent with the data from the literature, and could be explained by the sexual habits of younger women and by the fact that they have less immune experience than older women [48,49].

We found statistically significant differences in the HPV prevalence rates observed at different ages, a particularly high prevalence being noted in the first years of sexual activity. In the subsequent age-classes, the prevalence declined and stabilized. These results are supported by substantial data that indicate that the first HPV infections often occur soon after the first sexual intercourse [50-53]. In our adolescent group (16–17 years old), the prevalence of low-risk HPV infections was higher than that of probable/possible risk and high-risk HPV genotypes. The only multiple infection caused exclusively by HPV low-risk genotypes (HPV-6 and HPV-11 together) was detected in a 16-year-old. We observed a decrease in low-risk HPV infections with age, a finding that has been confirmed by other studies [54-56]. This decrease is consistent with the scant capacity of these genotypes to circumvent human defences. Several studies have reported a lower rate of persistence of infections by low-risk genotypes than of infections by high-risk types, suggesting that high-risk genotypes may more effectively evade the immune response and persist for a longer time in the human body [57-59]. Indeed, Franco et al. found that the mean duration of infections was 8.2 and 13.5 months for non-oncogenic and oncogenic types, respectively [60]. Moreover, in a study conducted on female university students, Richardson et al. found that infection by HPV-16 was the most persistent (mean duration 18.3 months), while HPV-6 displayed the shortest mean duration (< 10 months) [61]. The ability of high-risk HPV types to persist for a longer time in the host could be linked to the increase in the prevalence of high-risk HPV genotypes with age in our study.

Regarding the estimated cumulative proportions of young females without cervical lesions who were free from any HPV infection at each age, it is interesting that 49.8% of subjects were free from any HPV infection at the age of 26 years (Figure 4). Furthermore, if we consider the estimated cumulative proportions of females free from high-risk vaccine HPV infection at each age, 93.3% of 26-year-old women proved to be free from HPV-16 and/or HPV-18 (Figure 5A). Since the currently available HPV vaccines have shown partial cross-protection against high-risk oncogenic types [24,25], we also calculated the cumulative proportion of women free from infection by apha7/apha9 high-risk HPV species. At the age of 26 years, 92.2% and 84.7% of women, respectively, were also free from these infections (Figure 5B and 5C). In addition, at the age of 26 years, 97.1% of the women studied were free from infection by types 6 and 11 (Figure 5D).

In Italy, HPV vaccination has been offered free of charge to 12-year-old females since 2008, and both the quadrivalent and the bivalent vaccine are available. More recently, some Italian Regions have extended free vaccination to other female cohorts. Although the Italian Ministry of Health initially recommended vaccination for women aged 25–26 years, too [62], most Italian Regions offer vaccination free of charge only to younger women, with the aim of obtaining the maximum cost/effect benefit of vaccination in terms of public health [63]. The Italian strategy is similar to the strategies adopted by other developed countries and the member states of the European Union, despite obvious differences [30,64].

In this perspective, our study suggests that vaccinating females without cervical lesions up to 26 years of age appears to be useful both from the point of view of public health and from the point of view of the individual health of the women. Furthermore, studies on HPV vaccines have demonstrated that vaccination is effective, albeit to different degrees, both in naïve females and in women with evidence of prior HPV exposure [25].

Our considerations regarding the need to extend free vaccination to women up to 26 years of age are supported by epidemiological and economic studies. Epidemiological studies have shown that the risk of HPV infection remains in older age-groups; indeed, all sexually active women are exposed to the risk of infection at any age [27,65]. A meta-analysis on worldwide HPV prevalence in women with normal cytology has shown that HPV prevalence is high up to 34 years of age, subsequently decreases up to the age of 44 years, and then tends to rise again in older age-groups [27]. Moreover, economic evaluations also support the utility of extending free vaccination to women up to 26 years of age. Indeed, a study conducted in Italy has reported that vaccinating 25-year-old women is cost-effective, and that this strategy, in comparison with screening alone, could avoid 696 cases of cervical cancer, 11,000 cases of CIN1 and 1,500 cases of CIN2/3 [65]. Furthermore, Westra et al. developed a Markov model to estimate the age-specific health benefits and cost-effectiveness of vaccination in the Netherlands; they conclude not only that vaccinating girls against HPV before their sexual debut is a highly effective and cost-effective strategy for the prevention of cervical cancer, but also that vaccination of women up to the age of 25 years is generally cost-effective [66]. Finally, a review that reports the evidence for the dual approach of HPV vaccination and HPV-based cervical screening has reported that HPV vaccination is likely to provide additional protection to women under the age of 30 years [67].

## Conclusions

Our study confirms the importance of type 16 in both single and multiple infections and highlights the importance of type 52, which was second only to type 16 in women without cytological damage. Furthermore, it confirms the crucial importance of conducting studies on women without cytological damage, in order to optimise and up-date preventive interventions against HPV infection, such as vaccination.

Our findings also emphasize the importance of vaccinating young women up to 18 years of age through catch-up campaigns. Furthermore, they suggest the utility of updating vaccination strategies so as to include women up to 26 years of age, while taking the opportunity to perform a Pap test at the time of administering the first dose of vaccine. Nevertheless, this strategy should be further explored. This strategy could help to achieve good coverage, raise awareness of cervical cancer prevention and monitor the effectiveness of the vaccine at subsequent screening visits.

## Abbreviations

HPV: Human papillomavirus; IARC: International Agency for Research on Cancer; HR: High-risk; LR: Low-risk; LHU: Local Healthcare Unit; PBS: Phosphate buffered saline; NT: Not typing; CI: Confidence interval.

## Competing interests

The authors declare that they have no competing interests.

## Authors’ contributions

RG coordinated and supervised the research. RG, DP, ET and CMZ designed the study. RG and DP coordinated the Genoa unit’s research. ET coordinated the Milan unit’s research and the laboratory activities. CMZ coordinated the Turin unit’s research. SB and ERF carried out the laboratory analyses and performed the first data-quality control. PLL, AB and DA optimized the informatics database and performed the final data-quality control. RG and DP performed the first statistical analyses. SR supervised the first statistical analyses and conducted the final statistical analyses. RG, DP, DA and SR evaluated the results. RG, DP and DA wrote the manuscript. All authors revised the manuscript and contributed to improving the paper. All authors read and approved the final manuscript.

## Pre-publication history

The pre-publication history for this paper can be accessed here:

http://www.biomedcentral.com/1471-2334/13/575/prepub
